# Workplace bullying among employees in Germany: prevalence estimates and the role of the perpetrator

**DOI:** 10.1007/s00420-018-1366-8

**Published:** 2018-11-02

**Authors:** Stefanie Lange, Hermann Burr, Paul Maurice Conway, Uwe Rose

**Affiliations:** 1 Federal Institute for Occupational Safety and Health (BAuA), Nöldnerstraße 40-42, 10317 Berlin, Germany; 2 University of Copenhagen, Øster Farimagsgade 2A, 1353 Copenhagen, Denmark

**Keywords:** Bullying, S-MGA, Random sample, Workplace aggression, Harassment

## Abstract

**Objectives:**

The aim of this study was to examine the prevalence of workplace bullying in Germany while also taking the perpetrator and severity level (measured by frequency) into account and considering the role of gender, age and socio-economic status.

**Methods:**

We used data from a large representative sample (*N* = 4143) of employees in Germany subject to social security contributions. Self-reported bullying was assessed for different combinations of perpetrators (co-workers, superiors) and according to severity, i.e., being exposed at all and to severe bullying (at least weekly).

**Results:**

Prevalence estimates varied from 2.9% for severe bullying by co-workers to 17.1% for overall bullying (i.e., without distinguishing by perpetrator, less severe bullying also included). Unskilled workers reported more bullying by both perpetrators than academics/managers. We also observed an age trend for severe bullying by superiors (i.e., bossing), with younger employees being more affected from bossing than elder. No gender differences were detected.

**Conclusions:**

The findings indicate that it is crucial to consider type of perpetrator and severity of the behaviors when examining the prevalence of workplace bullying. The way bullying is defined and operationalized strongly contributes to the prevalence estimates. Differences between subgroups and associations or cause–effect relationships should be analyzed with these variations in mind.

## Introduction

Workplace bullying has been found to be a serious risk factor for a number of outcomes. These include negative effects on the physical and mental health of the targets (Bonde et al. 2016; Harvey et al. 2017; Kivimaki et al. 2003; Nielsen et al. 2014; Rugulies et al. 2012; Verkuil et al. 2015) and negative repercussions for the companies in terms of reduced performance, more absenteeism, and increased turnover (Glambek et al. 2014; McTernan et al. 2013; Nabe-Nielsen et al. 2017; Nielsen et al. 2016). The social security system is also affected as bullying is a risk factor for long-term sick leave, unemployment or early-retirement due to inability to work (Aagestad et al. 2014; Glambek et al. 2014).

A crucial starting point to adequately assess possible risks and develop courses of actions and recommendations is to achieve a sound knowledge about the extent of the bullying phenomenon. Estimating the prevalence of bullying is, however, difficult as workplace bullying is neither defined nor is operationalized in consistent ways (Kemp 2014). While there is general agreement that bullying takes place if an employee is persistently and repeatedly exposed to inappropriate treatment by one or more persons (Einarsen et al. 2011), the frequency of those treatments (i.e., the severity), the type of perpetrator, and whether the intention of the perpetrator or the experience of being a victim are necessary features of the definition are still debated issues (Hershcovis and Barling 2007).

Based on different understandings of the phenomenon, studies used different scaling methods and different criteria for classifying those exposed to negative behaviors as victims or non-victims of bullying (Conway et al. 2018; Notelaers and Einarsen 2013). Accordingly, it is not surprising that prevalence estimates of workplace bullying differ between studies and, owing to this, are difficult to compare (Nielsen et al. 2010). For example, there are studies revealing a prevalence ranging from 1.4% in Great Britain (Hoel et al. 2001) to 48% bullied employees in Turkey (Bilgel et al. 2006). Even within the same country, estimates may vary considerably; for instance, in Germany the prevalence of bullied persons ranged from 2.7% (Meschkutat et al. 2005) to 10.8% (zur Mühlen et al. 2001).

Some researchers have already attempted to explain the differences between prevalence estimates found in studies on grounds of the heterogeneous definitions and operationalizations of bullying. In particular, a meta-analysis by Nielsen et al. (2010) revealed that the prevalence estimates differed depending on whether workplace bullying was measured applying the self-labelling method or the behavioral experience method. For the self-labelling method, a single item is used asking about the participant’s exposure to bullying with a definition of bullying presented on beforehand, while the behavioral experience method (e.g., Negative Acts Questionnaire, Einarsen and Raknes 1997) requires respondents to rate their own exposure to a list of negative behaviors without these being explicitly referred to as instances of bullying. Nielsen et al. found that the prevalence estimates were systematically lower when using the self-labelling method with a definition (average prevalence 11.3%) than the behavioral experience method (average prevalence 14.8%). In a few cases the self-labelling method is used but where respondents were not presented to a definition of bullying before reporting if they were bullied (Niedhammer et al. 2012).

With regard to the behavioral experience method, Leymann (1996) proposed to classify targets of bullying based on three dimensions, that is the number, the frequency (i.e., severity) and the duration of the acts. Specifically, Leymann established that a responded should be considered as target of bullying if he/she reports at least one negative behavior occurring once a week for at least 6 months. As mentioned above, however, without an agreed definition of bullying, each of the three dimensions (number, severity, and duration) was treated differently in studies of bullying (Zapf et al. 2011). Previous research has shown that the narrower the criteria, the lower the prevalence estimates of self-reported bullying (Zapf et al. 2011). For example, Nielsen et al. (2009) showed that the range of prevalence estimates depended on the number of negative acts (only one act: 14.3%; at least two acts: 6.2%) and the severity (frequency of acts weekly: 0.6%; now and then: 4.6%). Furthermore, a longer reference period (12 months or lifetime exposure) increases the prevalence estimates of bullying (Schat et al. 2006). These patterns were confirmed in a review of 87 European studies (Zapf et al. 2011).

Nielsen at al. (2010) also showed that the prevalence estimates obtained in non-random samples (average prevalence 15.5%) are higher than those obtained in random samples (average prevalence 9.3%) when the self-labelling method with definition is used, while there are no differences when applying the behavioral experience method (non-random samples: 14.5%; random samples: 14.4%). Depending on the applied method, prevalence estimates in specific occupational sectors may not be generalized to other working populations. In addition, cultural differences may prevent from generalizing the prevalence estimates from one country to another. For example, the prevalence of bullying in Scandinavian countries seems to be systematically lower than in other countries, both within and outside Europe (Einarsen 2000; Zapf et al. 2011).

Furthermore, the type of perpetrator of the bullying acts is an important issue to consider when estimating the prevalence of bullying. There are usually more employees bullied by superior (so-called bossing) than by co-workers (Zapf et al. 2011), which results from the fact that the presence of an imbalance of power between targets and perpetrators is a central feature of bullying (Einarsen et al. 2003; Nielsen et al. 2011). Most studies, however, do not distinguish between sources of bullying, i.e., type of perpetrator. This is unfortunate, because different types of perpetrator may affect the targets dissimilarly. For example, a study demonstrated that employees bullied by superiors had a lower mental health than those bullied by colleagues and clients (Török et al. 2016). In previous studies, to probe the source of bullying participants were asked who the perpetrator was after participants labelled themselves as bullied (e. g. Mikkelsen and Einarsen 2001; Salin 2001). In one study, separate questions were asked for each type of perpetrator, but only with reference to ‘unpleasant situations’ and ‘aggression’ and not explicitly to bullying (Hubert and van Veldhoven 2001).

The present study sets out to address these gaps by investigating the prevalence of self-reported bullying among employees in Germany, taking both the source of bullying (co-workers, superiors, and without distinguishing by source) and the temporal dimension into account. With regard to the latter, we consider both the duration (i.e., how long) and the repetition (i.e., how often) of the bullying acts, being common elements in most definitions of bullying when assessing severity (Einarsen et al. 2011). To date, no studies have compared the prevalence of bossing and bullying by co-workers without the question about the source of the mistreatment being filtered by a previous overall rating of self-reported bullying.

In addition, we aimed at investigating prevalence estimates in relation to age, gender and socio-economic status. The influence of these factors on the prevalence of bullying enacted by distinct types of perpetrator and regarding the severity of bullying, i.e., its frequency, is poorly understood in current research. Finally, studies on bullying prevalence based on a nation-wide, random sample of employees in Germany are lacking.

## Materials and methods

### Population

Data was taken from the Study on Mental Health at Work (S-MGA), a nation-wide representative panel study with the first assessment taking place in 2011/2012. This study was initiated and funded by the Federal Institute of Occupational Safety and Health (BAUA), in cooperation with the Institute for Employment Research (IAB). S-MGA is the first study to examine the prevalence of self-reported workplace bullying using a random sample of employees in Germany with a face-to-face method of interviewing (computer-assisted personal interviewing, CAPI).

The population consisted of all employees subjected to social security contributions (without civil servants, self-employed individuals and freelancers) on the reference date of 31 December 2010. Using a two-stage cluster sampling procedure, 206 municipalities in Germany were randomly selected and then a gross sample of 13,590 addresses was drawn from them. The municipalities were stratified by region and population size.

The sample included in this study consisted of 4511 employees aged 31–60 years subject to social security contribution in the first wave of S-MGA in 2011/2012 (response rate: 35.7%). The S-MGA study does only cover this age range as the intention of the study was to select an age range of the German adult population with high labor market participation. The design and sampling procedure of S-MGA is described in more detail in Rose et al. (2017). Among the 4511 participants, 4182 (92% of the total sample) were employees. For the following analyses, we used all participants who were employees and did not have missing values for gender, age and the items used to measure bullying (*N* = 4143, corresponding to 99% of the eligible population). The distribution of gender, age and occupational status did not change after excluding those participants with missing values (Table 1).

**Table 1:** Distributions of gender, age and occupational status for employees, with and without missing values

	Employees			Employees without missing values (analysis sample)		
	*N*	%	95% CI	*N*	%	95% CI
Gender						
Men	2092	50	48–51	2068	49	48–51
Women	2089	50	45–51	2074	50	48–51
Age ( years)						
31–40	1131	27	25–28	1123	27	25–28
41–50	1776	42	40–44	1762	42	40–44
51–60	1273	30	29–31	1257	30	28–31
Occupation						
Unskilled workers	324	8	7–9	320	8	7–9
Skilled workers	1983	47	46–49	1965	47	46–49
Semi-professionals	1046	25	24–26	1038	25	24–26
Academics/managers	826	20	19–21	819	20	19–21
Total	4182	100		4143	100	

*N* = 4182

### Measures

Workplace bullying was assessed by the following four questions: (1) “Do you frequently feel unjustly criticized, hassled or shown up in front of others by co-workers?” and (3) “Do you frequently feel unjustly criticized, hassled or shown up in front of others by superiors?”, with the response options “yes” and “no”. Each of these two questions was followed by the question: (2, 4) “And how often did it occur in the last 6 months?” with the response options “daily”, “at least once a week”, “at least once a month” and “less than once a month”. Question (2) refers to co-workers and question (4) to superiors.

Based on these four questions we created the following six measures: bullying by co-workers and bossing, severe bullying by co-workers and severe bossing, overall bullying and severe overall bullying. For identifying cases of severe bullying, we applied the cutoff proposed by Leymann (1996), that is, self-reported exposure to bullying once a week for at least 6 months. Table 2 explains the classification based on perpetrator and severity.

**Table 2:** Classification of bullied employees based on perpetrator and severity

	Perpetrator		
	Bullied by co-workers	Bossing	Overall bullying
Severity			
Bullying	“Yes” to question (1)	“Yes” to question (3)	“Yes” to question (1) or (3)
Severe bullying	“Yes” to question (1) and responding “daily” or “at least once a week” to question (2)	“Yes” to question (3) and responding “daily” or “at least once a week” to question (4)	“Yes” to either severe bullying by co-workers or severe bossing or both

Question wordings:

(1) “Do you frequently feel unjustly criticized, hassled or shown up in front of others by co-workers?”

(2) “And how often did it occur in the last 6 months?”

(3) “Do you frequently feel unjustly criticized, hassled or shown up in front of others by superiors?”

(4) “And how often did it occur in the last 6 months?”

As one can see above—following Garthus-Niegel et al. (2016), bullying was thus assessed using a hybrid approach combining the behavioral experience and self-labelling methods. Specifically, the S-MGA had a single item without the global term “bullying” or a definition of the phenomenon being included; instead, the S-MGA added two examples of negative behaviors (i.e., “unjustly criticized”, “shown up in front of others”—German: “zu Unrecht kritisiert”, “vor anderen bloßgestellt”) and a specific type of self-labelling kind of bullying (“hassled”—German: “schikaniert”). Within the Gutenberg Health study, Garthus-Niegel and colleagues showed that this scaling method had the same predictive validity as the reporting of negative acts based on the behavioral experience method. Thus, it is a kind of parsimonious method which also addresses aspects of subjective experiences of being bullied by mentioning “to be hassled”.

For the present study, however, four changes were made to the original question by Garthus-Niegel et al. (2016). First, the sources of bullying were distinguished using different items for probing bullying perpetrated by superiors or co-workers to consider the relational or power issue. This enabled us to directly compare the prevalence estimates for different perpetrators instead of calculating the prevalence by source after an overall rating of bullying was provided by the respondents. Second, participants’ reports of being bullied—questions (1) and (3)—were separated from frequency of exposure using different questions—(2) and (4)—with different response categories. This allowed us to observe how the prevalence estimates changed when applying a criterion of frequency (i.e., severity) which matches the definitional component “repeatedly”. Third, a time frame of 6 months was integrated in the questions (2) and (4) to address the temporal component “persistently” and the suggested duration of (Leymann 1996). Finally, the response categories of questions (2) and (4) were changed from temporally vague frequency categories (always, often, sometimes, seldom, [almost] never) to more concrete labels as used by Leymann (e.g., daily, weekly, monthly).

#### Gender, age and socio-economic status

The computer-assisted personal interview included socio-demographic information on age, gender and occupation. Socio-economic status was assessed by occupational level inspired by Goldthorpe’s class theory (Goldthorpe 2000). Occupations were manually coded according to the International Standard Classification of Occupations (ISCO 08) and categorized into four groups on the basis of skill levels: unskilled workers, skilled workers, semi-professionals, academics/managers (Hagen 2015). Managers were included in the same group as professionals.

### Statistical analysis

To investigate prevalence estimates of workplace bullying, frequency analyses were carried out calculating confidence intervals (95%) based on sample weights. The latter were used to compensate for differences in the distribution of characteristics among the participants and the population (Schroder et al. 2015).

To examine differences by gender, age and socio-economic status, Chi-square tests were applied. All statistical analyses were conducted using IBM SPSS Statistics 24.

## Results

### Prevalence of self-reported bullying by perpetrators and severity

When disregarding severity (i.e., not applying any criterion of frequency), the prevalence of overall bullying was 17.1% in our study (Table 3). Bullying by co-workers (7.3%) occurred considerably less than bossing (13.3%). The prevalence estimates decreased by more than the half when applying a criterion of severity, for overall bullying (6.7%), for bullying by co-workers (2.9%) and bossing (5.0%).

**Table 3:** Prevalence estimates of bullying, overall and by type of perpetrator, and with and without a criterion of severity

	Bullied by co-workers		Bossing		Overall bullying^a^	
	%	95% CI	%	95% CI	%	95% CI
Bullying^b^	7.3	6.5–8.2	13.3	12.3–14.5	17.1	15.9–18.3
Severe bullying^c^	2.9	2.4–3.5	5.0	4.3–5.8	6.7	5.9–7.5

*N* = 4143

^a^At least one type of perpetrator (superiors, co-workers)

^b^At least once

^c^At least weekly within the last 6 months

When no criterion of severity was applied, among all employees who reported being bullied, 42% were bullied by co-workers (7.3% out of 17.1%) and 77% were bullied by superiors (13.3% out of 17.1%). The sum was larger than 100% because of an overlap between bullying from the two types of perpetrators. Table 4 shows the proportion of non-severely bullied employees (93.3%), employees reporting severe bullying exclusively by superiors (3.8%) or co-workers (1.6%), and participants reporting severe bullying by both perpetrators at the same time (1.2%). Among all employees being severely bullied by one type of perpetrator, the fraction of those reporting being bullied exclusively by their co-workers was lower (55%; 1.6% out of 2.9%) than the fraction of those reporting being bullied exclusively by their superiors (76%; 3.8% out of 5.0%).

**Table 4:** Proportion of severe bullying by perpetrator

	%	95% CI
Not severely bullied^a^	93.3	92.5–94.1
Severely bullied^b^ by superiors only	3.8	3.2–4.5
Severely bullied^b^ by co-workers only	1.6	1.3–2.1
Severely bullied^b^ by both	1.2	0.9–1.7
Total	100	

*N* = 4143

^a^Not bullied or bullied less than weekly within the last 6 months

^b^At least weekly within the last 6 months

### Prevalence of self-reported bullying by gender

We found no gender difference for any combination of perpetrator type and severity (Table 5). Point estimates for women were in all cases within the confidence intervals for men. The higher prevalence of bossing and the lower prevalence of bullying when using a criterion of severity were consistent among both men and women.

**Table 5:** Prevalence estimates by perpetrators, severity and gender

	Bullied by co-workers		Bossing		Overall bullying^a^	
	%	95% CI	%	95% CI	%	95% CI
Bullying^b^ (both genders)	7.3	6.5–8.2	13.3	12.2–14.5	17.1	15.9–18.3
Men	7.0	5.9–8.3	13.4	11.8–15.0	16.7	15.1–18.5
Women	7.7	6.5–9.0	13.3	11.8–14.9	17.4	15.7–19.2
Severe bullying^c^ (both genders)	2.9	2.4–3.5	5.0	4.3–5.8	6.7	5.9–7.5
Men	2.8	2.1–3.6	5.0	4.1–6.1	6.6	5.5–7.8
Women	3.0	2.3–3.9	5.0	4.1–6.1	6.7	5.7–8.0

*N* = 4143

^a^At least one type of perpetrator (superiors, co-workers)

^b^At least once

^c^At least weekly within the last 6 months

### Prevalence of self-reported bullying by age

**Fig. 1:**
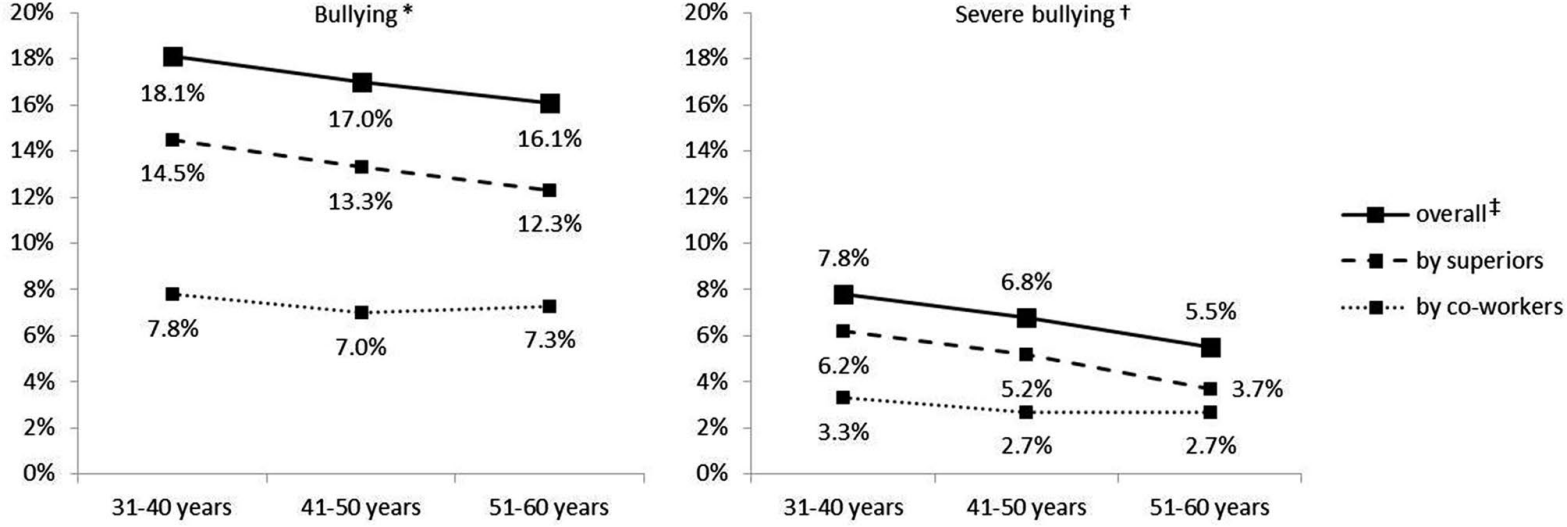
Prevalence estimates by perpetrator, severity and age. *N* = 4143. *At least once; ^†^At least weekly within the last 6 months; ^‡^At least one type of perpetrator (superiors, co-workers)

When a severity criterion was not applied, there were no significant differences between the three age groups for both bullying by co-workers and bossing (Fig. 1). When focusing on only severe bullying, older employees (51–60 years: 3.7%) reported significantly less bossing than their younger counterparts (31–40 years: 6.2%; 41–50 years: 5.2%; *χ*^2^ = 7.89, *p* = 0.032). Without distinguishing for perpetrators, however, this difference was not statistically significant, regardless of whether a criterion of severity was used (*p* = 0.104) or not (*p* = 0.474).

### Prevalence of self-reported bullying by socio-economic status

**Fig. 2:**
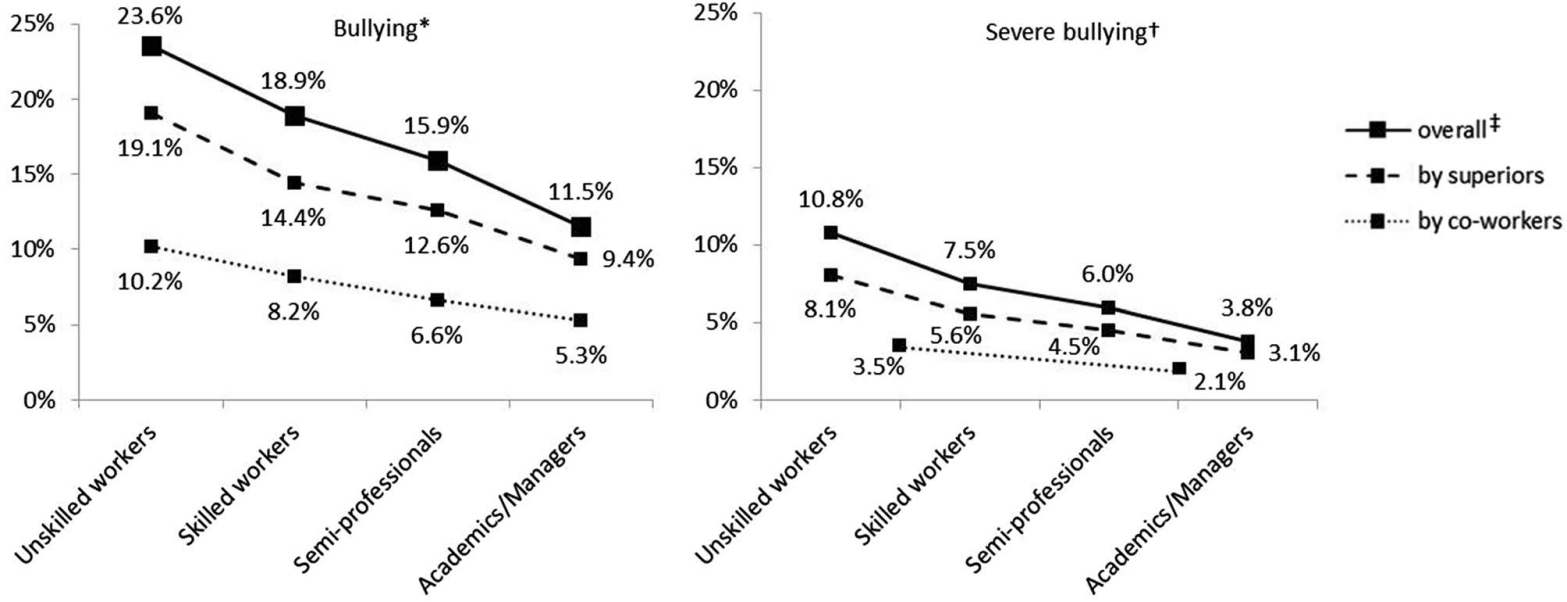
Prevalence estimates by perpetrator, severity and socio-economic status. *N* = 4143. *At least once; ^†^At least weekly within the last 6 months; ^‡^At least one type of perpetrator (superiors, co-workers). Regarding severe bullying by co-workers, for reasons of data protection the lower two and the upper two categories of socio-economic status were grouped

The prevalence estimates of bullying were significantly lower with higher socio-economic status (Fig. 2). This was the case for both types of perpetrators and for bullying with and without criterion of severity. The differences were more pronounced for bossing (bullying: *χ*^2^ = 22.70, *p* < 0.001; severe bullying: *χ*^2^ = 15.08, *p* = 0.004) than for bullying by co-workers (bullying: *χ*^2^ = 11.65, *p* = 0.018; severe bullying: *χ*^2^ = 7.23, *p* = 0.010). The already known trend (higher estimates for bossing than for bullying by co-workers and higher estimates for bullying than severe bullying) remained.

## Discussion

In the present study, we aimed at examining the prevalence of self-reported workplace bullying in Germany according to the source of the mistreatment and severity. This approach takes power relations and temporal issues into account, both of which are central to the understanding of bullying. Our results show that prevalence estimates differ depending on these dimensions: indeed, bossing occurs more frequently than bullying by co-workers and applying a criterion of severity (i.e., being exposed at last weekly, indicating severe bullying) reduces prevalence estimates by more than half. While we did not find differences by gender, we could observe an age difference, which was significant when focusing on superiors as perpetrator and applying a criterion of severity, with younger employees suffering from severe bossing more than older employees. In contrast, the impact of the socio-economic status—with a lower status being associated with a higher prevalence estimate of bullying—was significant regardless of type of perpetrator and severity.

In the present study, we used a hybrid approach to measure workplace bullying, combining the behavioral experience and the self-labelling methods. This choice was made in line with studies suggesting to combine the two approaches, because while both have limitations, they may complement each other in providing a more comprehensive information on bullying (Mikkelsen and Einarsen 2001; Nielsen et al. 2011). The measurement method we used in the S-MGA was parsimonious, showing the same predictive validity as the report of behavioral experiences (Garthus-Niegel et al. 2016). Moreover, it included two items from the Negative Acts Questionnaire (Einarsen and Raknes 1997), one of the most common used inventory of bullying behaviors, tapping both personal (“shown up in front of others”) and work-related (“unjustly criticized”) features of bullying (Einarsen et al. 2009). In addition, the item used in this study contained one specific type of self-labelling kind of bullying, namely “hassled” (German: “schikaniert”; to harass someone through hindrances, to annoy; Kunkel-Razum et al. 2003).

### Prevalence of self-reported bullying by perpetrators and severity

Depending on how one considers type of perpetrator and severity (i.e., frequency), prevalence estimates of self-reported bullying in Germany ranged from 2.9% (severe bullying by co-workers) to 17.1% (no criterion of severity and no distinction by source). With regard to the afore mentioned 2.9%, the prevalence of workplace bullying in Germany lies between Scandinavian countries (mostly far below 2%, e.g., Hauge et al. 2009; Nielsen et al. 2009) and Ireland and the UK (5% and below, e.g., Einarsen et al. 2009; O’Connell et al. 2007).

### Prevalence of self-reported bullying by perpetrators

As argued by Hershcovis and Barling (2007), the relational dimension (i.e., the relation between the perpetrator and the target) is often missing in investigations of bulling. In most studies, where this issue was addressed, respondents were first asked if they felt bullied; if this was the case, the respondents, where then asked who the perpetrator of the bullying behaviors was. In contrast, in the S-MGA a different approach was used with specific items for each type of perpetrator. This approach allowed for detailed information on severity on each type of bullying from co-workers and bossing for those people who have been bullied by both types of perpetrators.

There are almost twice as much employees reporting being bullied by superiors than by co-workers. A similar pattern was found in the review of Zapf et al. (2011), while other studies in Germany and Ireland found an even distribution (Meschkutat et al. 2005; O’Connell et al. 2007). In contrast, Scandinavian studies usually report significantly less bossing compared to bullying by co-workers, which might be explained by the presence, in these countries, of less asymmetrical relationships among employees and their superiors and more feminine values (Einarsen 2000; Ortega et al. 2009).

Another finding of this study was that, if an employee reported severe bullying by his/her co-workers, he/she was more likely to also report severe bossing than the other way round. This indicates that bossing is mostly a singular phenomenon, while employees being bullied by their co-workers are likely to be additionally bullied by their superiors. Maybe there is a specific organizational climate that facilitates bullying behavior for co-workers and superiors, while bossing alone is rather based on specific characteristics of the superior and his or her management style. Assuming that the burden of being exposed to bullying from different perpetrators is higher, this group being bullied by both types of perpetrators needs focused and near-time support.

### Prevalence of self-reported bullying by gender

We did not find any gender differences among bullied employees, independent of the perpetrator or the severity. This corresponds to the results of Zapf et al. (2011), who found out that potentially higher prevalence estimates for women only reflected the gender composition in the examined sample. Since our sample is representative for employees in Germany subject to social security contribution, the gender distribution was even and, as a result, also the prevalence estimates of bullying were evenly spread.

Another explanation for gender differences found in other studies might be the industrial groups or organizations, where the studies took place. In male-dominated industries, women represent minority groups, which have been previously shown to be at higher risk of bullying (Hogh et al. 2011; Ortega et al. 2009).

### Prevalence of self-reported bullying by age

The prevalence of bossing seems to be more dependent on age than bullying by co-workers, but only for severe bullying. Younger employees reported more severe bossing than older employees, which is not in line with the results of some studies from Scandinavia (Einarsen and Skogstad 1996; Lahelma et al. 2012; Oxenstierna et al. 2012) or Ireland (O’Connell et al. 2007), where older employees were rather more affected by bullying. Previous studies in Germany revealed different results. While Garthus-Niegel et al. (2016) found no significant association with age regarding overall bullying—as we did—, Meschkutat et al. (2005) reported a u-shaped age relation: the most affected group consisted of participants aged below 25, followed by participants older than 54 years. Participants aged between 25 and 54 reported less bullying experience. Although we also found higher estimates of prevalence for younger employees as Meschkutat et al. (2005), the S-MGA did not include people below 31 years. Thus, the age group with the highest prevalence estimate in our study (31–40 years) was one of the groups (25–34 years, 35–44 years) with lower prevalence estimate in the study of Meschkutat et al. (2005). It would be of interest to know if a distinction by perpetrator and by severity would reveal similar age patterns in those other populations with older employees being less affected by severe bossing.

As mentioned above, the existence of an imbalance of power between the target and the perpetrator is often conceived as an important feature in the definition of bullying (Einarsen et al. 2003). Younger employees usually have little power, whereas the superior is probably not only older but has also a longer seniority, which may result in a substantial power imbalance. From this point of view, our findings make sense. Thus, it might be that experience in the labor market rather than biological age is crucial for being bullied, because both characteristics are closely related.

### Prevalence of self-reported bullying by socio-economic status

In our study we additionally found that the higher the socio-economic status, the lesser the prevalence estimates of self-reported bullying – regardless of the perpetrator or the severity. This in line with the results of Ortega et al. (2009), who considered occupational status (unskilled workers, skilled workers, salaried staff and public servants, managers/supervisors) and found that unskilled workers reported more bullying than employees in higher occupational categories.

The power issue might again explain why unskilled workers reported being bullied most frequently, while managers or academics reported the least frequent exposure. With a lower socio-economic status, influence at work is lower (Landsbergis et al. 2014; Moncada et al. 2010), a condition that, as mentioned above, is associated with a higher risk of becoming a target of bullying (Einarsen et al. 2003; Nielsen et al. 2011). This is substantiated by our result that the impact of socio-economic status is stronger for bossing than for bullying by co-workers; an explanation for this finding could be that, with a higher socio-economic status, the power distance between superiors and subordinates decreases.

Our findings regarding especially socio-economic status might be explained by other individual characteristics such as personality (Chapman et al. 2010). The influence of certain personality traits on the prevalence of bullying is less investigated; S-MGA has not measured personality either. As Nielsen and Knardahl (2015) showed in their longitudinal study, personality traits can be both, predictors and outcomes of bullying, so our cross-sectional analyses for this study would not have been suitable for investigating the role of personality. Future longitudinal studies focusing on the antecedents of bullying should take personality into account.

### Strengths and limitations

Since bullying is not widespread among employees, studies with small sample sizes have limited power when it comes to investigating subgroups. On the contrary, the S-MGA sample is large enough to provide reliable estimates of bullying prevalence by gender, age and socio-economic status. Next to the sample size, S-MGA is based on a random sample which allows reliable estimations of bullying by socio-demographic characteristics. In contrast, non-random samples may have limitations when it comes to estimating the prevalence of bullying of subgroups. Our results are, however, not representative for employees younger than 31 and for employees who are not subjected to social security contribution, because both were excluded given the sampling procedure. We do not know to what extent the prevalence estimates of workplace bullying within the excluded subgroups would differ from the investigated sample; except for those subgroups, however, the present sample can be generalized to all employees in Germany.

In the present study, we have studied employees aged 31 years and above. It would have been of interest to know the prevalence of bullying among people in the 20s, but as the S-MGA did only include older employees it was not possible for us to do that.

In the S-MGA, the possible perpetrators were limited to co-workers and superiors, while subordinates or other perpetrators such as clients, costumers, students, pupils or patients were not considered. The share of employees reporting being bullied by those perpetrators is usually relatively small, for example 5% in a Danish population (Rugulies et al. 2012) and 10% among all targets according to a review (Zapf et al. 2011). Still, future studies should consider adding further items regarding other perpetrators to get a complete picture, especially if specific settings as schools or hospitals are examined.

We could not consider personality as a predictor of bullying victimization as this was not included in the questionnaire. The literature has focused less on personality traits than on external factors and in the few studies looking at personality, the role thereof is unclear in the victimization process.

Finally, the mode of data collection in the present study might have influenced the prevalence estimates. While the large majority of studies in the field used methods not involving an interviewer such as paper-and-pencil questionnaires or online-assisted interviewing, we conducted personal interviews. Thus, we cannot exclude that an underreporting of bullying occurred due to the face-to-face interview setting employed (Feveile et al. 2007; Krumpal 2013). Unfortunately, we cannot predict the size of this possible bias, as to our knowledge no studies on prevalence of bullying have investigated the effect of mode of data collection.

## Conclusions and implications

Using a hybrid measurement approach combining the self-labelling and the behavioral experience methods, in the present study we provided different prevalence estimates of self-reported bullying, ranging from 2.9 to 17.1% depending of the source and severity (i.e., frequency of behaviors), in a large representative sample of employees in Germany subject to social security contribution. The prevalence estimates were higher when a stricter criterion was used (i.e., being exposed at least weekly, corresponding to severe bullying) and when the source of bullying were superiors instead of co-workers. In line with the existing literature, we did not find any gender differences in the prevalence of bullying. We found, however, a deviating pattern with regards to age. Specifically, the prevalence estimate of severe bossing was higher for younger employees than for older employees, while bullying by co-workers was evenly distributed between age groups. Hence, practical interventions against bullying should focus particularly on leadership and support to younger employees at the beginning of their career. As lower socio-economic status increased the likelihood of being bullied, interventions may also take this tendency into account. For example, organizational changes like flattening the hierarchies or increasing control might act as a protective factor.

From a methodological point of view, the role of different perpetrators and the severity should be always considered in studies on bullying. Our results indicate that the prevalence of bullying should be examined for different combinations of perpetrator and severity, to prevent over- or underestimating bullying for specific groups (e.g., targets of severe bossing). Finally, the variety of prevalence estimates is also important when investigating possible causes or consequences of bullying. If only one combination of perpetrator and severity is analyzed, it might be that relevant associations remain hidden.

## Appendix

Original items (response categories in parentheses):

(1) “Fühlen Sie sich durch Kollegen häufig zu Unrecht kritisiert, schikaniert oder vor anderen bloßgestellt?” (ja, nein).

(2) “Und wie häufig kam dies in den letzten 6 Monaten vor? War das…?” (täglich, mindestens einmal pro Woche, mindestens einmal pro Monat, seltener als einmal im Monat).

(3) “Nun zu Ihren Vorgesetzten. Fühlen Sie sich durch Vorgesetzte häufig zu Unrecht kritisiert, schikaniert oder vor anderen bloßgestellt?” (ja, nein).

(4) “Und wie häufig kam dies in den letzten 6 Monaten vor? War das…?” (täglich, mindestens einmal pro Woche, mindestens einmal pro Monat, seltener als einmal im Monat).

## References

[CR1] Aagestad C, Johannessen H, Tynes T, Magne Gravseth H, Sterud T (2014). Work-related psychosocial risk factors for long-term sick leave—a prospective study of the general working population in Norway. J Occup Environ Med.

[CR2] Bilgel N, Aytac S, Bayram N (2006). Bullying in Turkish white-collar workers. Occup Med.

[CR3] Bonde JP (2016). Health correlates of workplace bullying: a 3-wave prospective follow-up study. Scand J Work Environ Health.

[CR4] Chapman BP, Fiscella K, Kawachi I, Duberstein PR (2010). Personality, socioeconomic status, and all-cause mortality in the United States. Am J Epidemiol.

[CR5] Conway P (2018). Optimal CUT-off points for the short-negative act questionnaire and their association with depressive symptoms and diagnosis of depression. Ann Work Expo Health.

[CR6] Einarsen S (2000). Harassment and bullying at work: a review of the Scandinavian approach. Aggress Violent Behav.

[CR7] Einarsen S, Raknes B (1997). Harassment in the workplace and the victimization of men. Violence Vict.

[CR8] Einarsen S, Skogstad A (1996). Bullying at work: epidemiological findings in public and private organizations. Eur J Work Organ Psychol.

[CR9] Einarsen S, Hoel H, Zapf D, Cooper C, Einarsen S, Hoel H, Zapf D, Cooper C (2003). The concept of bullying at work: the European tradition. Bullying and emotional abuse in the workplace: international perspectives in research and practice.

[CR10] Einarsen S, Hoel H, Notelaers G (2009). Measuring exposure to bullying and harassment at work: validity, factor structure and psychometric properties of the Negative Acts Questionnaire-Revised. Work Stress.

[CR11] Einarsen S, Hoel H, Zapf D, Cooper CL, Einarsen S, Hoel H, Zapf D, Cooper CL (2011). The concept of bullying at work: the European tradition. Bullying and harassment in the workplace: developments in theory, research, and practice.

[CR12] Feveile H, Olsen O, Hogh A (2007). A randomized trial of mailed questionnaires versus telephone interviews: response patterns in a survey. BMC Med Res Methodol.

[CR13] Garthus-Niegel S (2016). Development of a mobbing short scale in the Gutenberg Health Study. Int Arch Occup Environ Health.

[CR14] Glambek M, Matthiesen SB, Hetland J, Einarsen S (2014). Workplace bullying as an antecedent to job insecurity and intention to leave: a 6-month prospective study. Hum Resour Manag J.

[CR15] Goldthorpe JH (2000). On sociology: numbers, narratives, and the integration of research and theory.

[CR16] Hagen F (2015) Levels of education: relation between ISCO skill level and ISCED categories. In: Telematic multidisciplinary assistive technology education. http://www.fernunihagen.de/FTB/telemate/database/isced.htm#ISCO. Accessed 21 Mar 2015

[CR17] Harvey S (2017). Can work make you mentally ill? A systematic meta-review of work-related risk factors for common mental health problems. Occup Environ Med.

[CR18] Hauge LJ, Skogstad A, Einarsen S (2009). Individual and situational predictors of workplace bullying: Why do perpetrators engage in the bullying of others?. Work Stress.

[CR19] Hershcovis MS, Barling J, Langan-Fox J, Cooper CL, Klimoski RJ (2007). Towards a relational model of workplace aggression. Research companion to the dysfunctional workplace: management challenges and symptoms.

[CR20] Hoel H, Cooper CL, Faragher B (2001). The experience of bullying in Great Britain: THE impact of organizational status. Eur J Work Organ Psychol.

[CR21] Hogh A, Carneiro IG, Giver H, Rugulies R (2011). Are immigrants in the nursing industry at increased risk of bullying at work? A one-year follow-up study. Scand J Psychol.

[CR22] Hubert AB, van Veldhoven M (2001). Risk sectors for undesirable behaviour and mobbing. Eur J Work Organ Psychol.

[CR23] Kemp V (2014). Antecedents, consequences and interventions for workplace bullying. Curr Opin Psychiatry.

[CR24] Kivimaki M, Virtanen M, Vartia M, Elovainio M, Vahtera J, Keltikangas-Järvinen L (2003). Workplace bullying and the risk of cardiovascular disease and depression. Occup Environ Med.

[CR25] Krumpal I (2013). Determinants of social desirability bias in sensitive surveys: a literature review. Qual Quant.

[CR26] Kunkel-Razum K, Scholze-Stubenrecht W, Wermke M (2003). Deutsches Universalwörterbuch [German universal dictionary].

[CR27] Lahelma E, Lallukka T, Laaksonen M, Saastamoinen P, Rahkonen O (2012). Workplace bullying and common mental disorders: a follow-up study. J Epidemiol Community Health.

[CR28] Landsbergis PA, Grzywacz JG, LaMontagne AD (2014). Work organization, job insecurity, and occupational health disparities. Am J Ind Med.

[CR29] Leymann H (1996). The content and development of mobbing at work. Eur J Work Organ Psychol.

[CR30] McTernan W, Dollard M, LaMontagne A (2013). Depression in the workplace: an economic cost analysis of depression-related productivity loss attributable to job strain and bullying. Work Stress.

[CR31] Meschkutat B, Stackelbeck M, Langenhoff G (2005). Der Mobbing-Report: eine Repräsentativstudie für die Bundesrepublik Deutschland.

[CR32] Mikkelsen EG, Einarsen S (2001). Bullying in Danish work-life: prevalence and health correlates. Eur J Work Organ Psychol.

[CR33] Moncada S (2010). Psychosocial work environment and its association with socioeconomic status. A comparison of Spain and Denmark. Scand J Public Health.

[CR34] Nabe-Nielsen K (2017). The role of psychological stress reactions in the longitudinal relation between workplace bullying and turnover. J Occup Environ Med.

[CR35] Niedhammer I, Sultan-Taïeb H, Chastang J-F, Vermeylen G, Parent-Thirion A (2012). Exposure to psychosocial work factors in 31 European countries. Occup Med.

[CR36] Nielsen MB, Knardahl S (2015). Is workplace bullying related to the personality traits of victims? A two-year prospective study. Work Stress.

[CR37] Nielsen M (2009). Prevalence of workplace bullying in Norway: comparisons across time and estimation methods. Eur J Work Organ Psychol.

[CR38] Nielsen M, Matthiesen S, Einarsen S (2010). The impact of methodological moderators on prevalence rates of workplace bullying. A meta-analysis. J Occup Organ Psychol.

[CR39] Nielsen M, Notelaers G, Einarsen S, Einarsen S, Hoel H, Zapf D, Cooper CL (2011). Measuring exposure to workplace bullying. Bullying and harassment in the workplace: developments in theory, research, and practice.

[CR40] Nielsen M, Magerøy N, Gjerstad J, Einarsen S (2014). Workplace bullying and subsequent health problems. J Nor Med Assoc.

[CR41] Nielsen M, Indregard A-MR, Øverland S (2016) Workplace bullying and sickness absence: a systematic review and meta-analysis of the research literature. Scand J Work Environ Health(5):359–370 10.5271/sjweh.3579 10.5271/sjweh.3579 27310716

[CR42] Notelaers G, Einarsen S (2013). The world turns at 33 and 45: defining simple cutoff scores for the Negative Acts Questionnaire-Revised in a representative sample. Eur J Work Organ Psychol.

[CR43] O’Connell P, Calvert E, Watson D (2007) Bullying in the workplace: survey reports 2007. Department of Enterprise, Trade and Employment

[CR44] Ortega A, Hogh A, Pejtersen JH, Feveile H, Olsen O (2009). Prevalence of workplace bullying and risk groups: a representative population study. Int Arch Occup Environ Health.

[CR45] Oxenstierna G, Elofsson S, Gjerde M, Magnusson Hanson L, Theorell T (2012). Workplace bullying, working environment and health. Ind Health.

[CR46] Rose U (2017). The study on mental health at work: design and sampling. Scand J Public Health.

[CR47] Rugulies R (2012). Bullying at work and onset of a major depressive episode among Danish female eldercare workers. Scand J Work Environ Health.

[CR48] Salin D (2001). Prevalence and forms of bullying among business professionals: a comparison of two different strategies for measuring bullying. Eur J Work Organ Psychol.

[CR49] Schat A, Frone M, Kevin Kelloway E (2006) Prevalence of workplace aggression in the U.S. Workforce: findings from a National Study

[CR50] Schroder H, Schiel S, Schulz S, Kleudgen M (2015) Mentale Gesundheit bei der Arbeit (S-MGA): Methodenbericht zur Repräsentativerhebung an Erwerbstätigen in Deutschland. Projekt F. 2., vol überarb. Aufl. ed. Bundesanstalt für Arbeitsschutz, Dortmund/Berlin/Dresden

[CR51] Török E, Hansen AM, Grynderup MB, Garde AH, Hogh A, Nabe-Nielsen K (2016). The association between workplace bullying and depressive symptoms: the role of the perpetrator. BMC Public Health.

[CR52] Verkuil B, Atasayi S, Molendijk ML (2015). Workplace bullying and mental health: a meta-analysis on cross-sectional and longitudinal data. PLoS One.

[CR53] Zapf D, Escartin J, Einarsen S, Hoel H, Vartia M, Einarsen S, Hoel H, Zapf D, Cooper CL (2011). Empirical findings on prevalence and risk groups of bullying in the workplace. Bullying and harassment in the workplace: developments in theory, research, and practice.

[CR54] zur Mühlen L, Normann G, Greif S (2001) Stress and bullying in two organisations. Faculty of Psychology. University of Osnabrück

